# Hyperuricemia is associated with decreased changes in heart rate variability after hemodialysis in non-diabetic patients

**DOI:** 10.18632/oncotarget.23981

**Published:** 2018-01-04

**Authors:** Po-Chih Chen, Pei-Yu Wu, Jiun-Chi Huang, Szu-Chia Chen, Yeou-Lih Huang

**Affiliations:** ^1^ Department of Medical Laboratory Science and Biotechnology, College of Health Sciences, Kaohsiung Medical University, Kaohsiung, Taiwan; ^2^ Department of Laboratory Medicine, Kaohsiung Medical University Hospital, Kaohsiung Medical University, Kaohsiung, Taiwan; ^3^ Graduate Institute of Clinical Medicine, College of Medicine, Kaohsiung Medical University, Kaohsiung, Taiwan; ^4^ Division of Nephrology, Department of Internal Medicine, Kaohsiung Medical University Hospital, Kaohsiung Medical University, Kaohsiung, Taiwan; ^5^ Department of Internal Medicine, Kaohsiung Municipal Hsiao-Kang Hospital, Kaohsiung Medical University, Kaohsiung, Taiwan; ^6^ Faculty of Medicine, College of Medicine, Kaohsiung Medical University, Kaohsiung, Taiwan; ^7^ Department of Chemistry, National Sun Yat-sen University, Kaohsiung, Taiwan

**Keywords:** heart rate variability change before and after hemodialysis, hemodialysis, non-diabetes, uric acid

## Abstract

Hyperuricemia has been associated with low heart rate variability (HRV), however whether there is an association between uric acid (UA) and HRV changes after hemodialysis (HD) is unknown. The aim of this study was to investigate the role of UA in HRV changes before and after HD in non-diabetic patients. Ninety-six non-diabetic patients under maintenance HD were enrolled. HRV was examined to assess changes before and after HD. A change in HRV (ΔHRV) was calculated as post-HD HRV minus pre-HD HRV. Compared to the patients with a UA level ≦ 7 mg/dL, those with a UA level > 7 mg/dL had lower ∆high frequency (HF)% (*p* = 0.027). UA was negatively associated with ∆HF% (*r* = -0.247, *p* = 0.015) and ∆low frequency (LF)/HF (*r* = -0.236, *p* = 0.021) in the non-diabetic patients undergoing HD. Furthermore, in multivariate analysis after adjustments for demographic, clinical, and biochemical characteristics and medications, UA was independently associated with ∆HF% (per 1 mg/dL, unstandardized coefficient β = -2.892; 95% CI, -5.066 to -0.717; *p* = 0.010) and ∆LF/HF (per 1 mg/dL, unstandardized coefficient β = -0.165; 95% CI, -0.291 to -0.038; *p* = 0.011). Hyperuricemia contributed to lesser HF% and LF/HF increase after HD in the non-diabetic patients, reflecting a state of impaired sympatho-vagal equilibrium in non-diabetic HD patients with hyperuricemia. Lowering UA levels may have the potential to improve increased HRV in non-diabetic HD patients.

## INTRODUCTION

Cardiovascular autonomic neuropathy can be evaluated according to variations in heart rate, termed heart rate variability (HRV). HRV is defined as variations in R-R interval and instantaneous heart rate on electrocardiography, and it has been shown to be a simple and noninvasive method to assess autonomic nervous system activity [1]. HRV can be categorized as being high-frequency (HF) and low-frequency (LF) [1]. HF HRV is analogous to respiratory sinus arrhythmia, and it can be used to characterize vagal control of the heart rate [2]. Both vagal and sympathetic activities have been reported to contribute to LF HRV [3], and normalized LF (LF%) and the LF/HF ratio have been reported to reflect sympatho-vagal balance and sympathetic modulation [1]. HRV (low LF and HF) has been shown to be drastically decreased in patients undergoing chronic hemodialysis (HD) compared to healthy controls, implying increased autonomic dysfunction and sympathetic activation [4]. In addition, low HRV has been associated with adverse cardiovascular outcomes in chronic HD patients [5–7]. Moreover, dialysis-induced changes are consistent with compensatory baroreflex-mediated activation of the sympathetic nervous system according to HRV in HD patients [8]. We recently reported that changes in HRV before and after HD (∆HRV) can be used to predict overall and cardiovascular mortality [9]. Zitt et al. [10] also evaluated the association between diabetes and autonomic cardiovascular regulation during HD. In their study, eight diabetic patients showed a blunted autonomic response, whereas HRV was found to be increased during dialysis in nine non-diabetic patients [8]. Impaired autonomic function may therefore be related to damage due to diabetes in autonomic neuropathy. Although a previous study investigated the factors related to ∆HRV in non-diabetic HD patients, it was limited by a small sample size [11].

Many epidemiological studies have reported that hyperuricemia is independently associated with increased mortality, cardiovascular disease, and renal disease in the general population [12, 13]. Hyperuricemia is common in patients with renal failure, possibly due to decreased renal excretion of uric acid (UA) in patients with impaired renal function. In addition, hyperuricemia has been associated with various risk factors for renal failure [12, 14, 15]. Previous studies have reported an association between hyperuricemia and low HRV parameters in various populations [16–18]. However, few studies have evaluated the relationship between UA and changes in HRV before and after HD. Therefore, the aim of this study was to assess the role of UA in HRV changes before and after HD in non-diabetic patients.

## RESULTS

The mean age of the 96 patients was 59.8 ± 12.2 years. Comparisons of baseline characteristics between the patients with a UA level ≦ 7 mg/dL and those with a UA level > 7 mg/dL are shown in Table 1. Compared to the patients with a UA level ≦ 7 mg/dL, those with a level > 7 mg/dL were younger, had higher levels of albumin, creatinine, and total calcium, and lower rates of β-blocker and calcium channel blocker use. In addition, the patients with UA > 7 mg/dL had a lower ∆high HF%.

**Table 1:** Comparison of baseline characteristics between non-diabetic hemodialysis patients with uric acid ≦ 7 mg/dL and > 7 mg/dL

Characteristics	All patients (*n =* 96)	With uric acid ≦ 7 mg/dL (*n =* 30)	With uric acid > 7 mg/dL (*n =* 66)	*P*
Age (year)	59.8 ± 12.2	64.4 ± 14.2	57.7 ± 10.6	0.012
Male gender (%)	36.5	36.7	36.4	0.977
Duration of dialysis (years)	8.4 (4.6–12.7)	7.8 (4.7–12.7)	8.7 (4.6–12.5)	0.650
Smoking history (%)	24.0	23.3	24.2	0.923
Hypertension (%)	50.0	56.7	47.0	0.378
Coronary artery disease (%)	17.7	16.7	18.2	0.857
Cerebrovascular disease (%)	3.1	3.3	3.0	0.937
Systolic blood pressure (mmHg)	144.1 ± 23.2	143.9 ± 22.7	144.1 ± 23.6	0.973
Diastolic blood pressure (mmHg)	79.9 ± 13.5	79.9 ± 12.2	79.9 ± 14.1	0.998
Laboratory parameters				
Albumin (g/dL)	3.9 ± 0.3	3.8 ± 0.2	3.9 ± 0.3	0.020
Fasting glucose (mg/dL)	98.2 ± 26.0	99.7 ± 24.0	97.5 ± 27.1	0.710
Triglyceride (mg/dL)	118 (87–199)	137 (91.3–189.5)	108 (85.5–201)	0.494
Total cholesterol (mg/dL)	188.1 ± 39.6	183.7 ± 35.9	190.2 ± 41.2	0.464
Hemoglobin (g/dL)	10.2 ± 1.0	10.2 ± 1.1	10.1 ± 1.0	0.843
Creatinine (mg/dL)	10.0 ± 2.2	8.7 ± 2.1	10.7 ± 2.0	< 0.001
Potassium (mEq/L)	4.5 ± 0.8	4.6 ± 0.9	4.5 ± 0.7	0.414
Total calcium (mg/dL)	9.5 ± 1.1	9.1 ± 1.2	9.7 ± 1.0	0.015
Phosphorous (mg/dL)	4.4 ± 1.0	4.4 ± 1.0	4.4 ± 1.0	0.808
CaXP product (mg^2^/dL^2^)	41.8 ± 10.4	39.4 ± 8.9	42.9 ± 10.9	0.136
iPTH (pg/mL)	391.1 (184.9–487.6)	392.9 (128.4–421.2)	385 (197.1–643.1)	0.579
Uric acid (mg/dL)	7.9 ± 1.6	6.3 ± 0.6	8.7 ± 1.4	< 0.001
Kt/V (Daugirdes)	1.6 ± 0.3	1.7 ± 0.4	1.6 ± 0.2	0.272
Ultrafiltration (%)	4.1 ± 1.4	4.1 ± 1.5	4.1 ± 1.4	0.985
∆HRV parameters (frequency domain)				
∆LF (ms^2^)	0.16 ± 0.47	1.06 ± 0.32	−0.25 ± 0.66	0.194
∆HF (ms^2^)	0.22 ± 0.37	0.51 ± 0.36	0.09 ± 0.52	0.606
∆LF% (nu)	−4.66 ± 2.3	−10.34 ± 4.58	−2.09 ± 2.61	0.099
∆HF% (nu)	2.06 ± 1.71	7.63 ± 3.67	−0.47 ± 1.78	0.027
∆LF/HF	0.22 ± 0.12	0.56 ± 0.23	0.07 ± 0.13	0.057
Medications				
ACEI and/or ARB use	13.6	14.8	13.1	0.830
β-blocker use	14.8	33.3	6.6	0.002
Calcium channel blocker use	18.2	37.0	9.8	0.002
Statins use	20.5	14.8	23.0	0.568

Abbreviations. CaXP product, Calcium-phosphorous product; iPTH, intact parathyroid hormone; HRV, heart rate variability; LF, low frequency; HF, high frequency; ACEI, angiotensin converting enzyme inhibitor; ARB, angiotensin II receptor blocker.

### Correlations between UA and ∆HRV parameters

Correlations between UA and ∆HRV parameters in the non-diabetic HD patients are shown in Table 2. UA was negatively associated with ∆HF% (*r* = −0.247, *p* = 0.015) and ∆LF/HF (*r* = −0.236, *p* = 0.021). However, UA was not correlated with ∆LF (*p* = 0.264), ∆HF (*p* = 0.890) or ∆LF% (*p* = 0.115).

**Table 2:** Correlation between uric acid and ∆HRV parameters in non-diabetic hemodialysis patients

∆HRV parameters	Pearson’s r	*p*
∆LF (ms^2^)	−0.115	0.264
∆HF (ms^2^)	−0.014	0.890
∆LF% (nu)	0.162	0.115
∆HF% (nu)	−0.247	0.015
∆LF/HF	−0.236	0.021

Values expressed as r. Abbreviations are the same as in Table 1.

### Determinants of ∆HF%

The multiple stepwise analysis Table 3 revealed that ∆HF% was independently correlated with potassium (per 1 mEq/L, unstandardized coefficient β = 6.868; 95% confidence interval [CI], 2.109 to 11.628; *p* = 0.005), total calcium (per 1 mg/dL, unstandardized coefficient β = 5.177; 95% CI, 1.776 to 8.578; *p* = 0.003) and UA (per 1 mg/dL, unstandardized coefficient β = −2.892; 95% CI, -5.066 to -0.717; *p* = 0.010) after adjusting for age, sex, hypertension, duration of dialysis, a history of smoking, coronary artery disease, cerebrovascular disease, systolic blood pressure, diastolic blood pressure, levels of albumin, fasting glucose, triglyceride, total cholesterol, hemoglobin, creatinine, potassium, total calcium, phosphorous, calcium-phosphorous product, intact parathyroid hormone (iPTH), and UA, Kt/V, percentage of ultrafiltration, and medications including calcium channel blockers, β-blockers, angiotensin converting enzyme inhibitors (ACEIs), angiotensin II receptor blockers (ARBs), and statins.

**Table 3:** Determinants of ∆HRV parameters of non-diabetic hemodialysis patients

∆HRV parameters	Multivariate (Stepwise)	
	Unstandardized coefficient β (95% CI)	*p*
∆HF%		
Potassium (per 1 mEq/L)	6.868 (2.109, 11.628)	0.005
Total calcium (per 1 mg/dL)	5.177 (1.776, 8.578)	0.003
Uric acid (per 1 mg/dL)	−2.892 (−5.066, −0.717)	0.010
∆LF/HF		
Potassium (per 1 mEq/L)	0.462 (0.185−0.739)	0.001
Total calcium (per 1 mg/dL)	0.284 (0.086–0.483)	0.005
Uric acid (per 1 mg/dL)	−0.165 (−0.291, −0.038)	0.011

Values expressed as unstandardized coefficient β and 95% confidence interval (CI). Abbreviations are the same as in Table 1.

Covariates in the multivariate model included age, sex, duration of dialysis, a history of smoking, hypertension, coronary artery disease and cerebrovascular disease, systolic and diastolic blood pressure, albumin, fasting glucose, triglyceride, total cholesterol, hemoglobin, creatinine, potassium, total calcium, phosphorous, CaXP product, iPTH, uric acid, Kt/V, ultrafiltration percent, and medications including ACEIs and/or ARBs, β-blockers, calcium channel blockers, and statins.

### Determinants of ∆LF/HF

The multiple stepwise analysis Table 3 revealed that ∆LF/HF was independently correlated with potassium (per 1 mEq/L, unstandardized coefficient β = 0.462; 95% CI, 0.185 to 0.739; *p* = 0.001), total calcium (per 1 mg/dL, unstandardized coefficient β = 0.284; 95% CI, 0.086 to 0.483; *p* = 0.005) and UA (per 1 mg/dL, unstandardized coefficient β = −0.165; 95% CI, −0.291 to −0.038; *p* = 0.011).

### Risk of hospitalization

The median and range of follow-up period was 27.3 (1.5−38) months for all patients. During the follow-up period, 49 hospitalizations were recorded among these 96 patients (51.0%), including CV events (*n* = 13), gastrointestinal disorders or bleeding (*n* = 5), infectious diseases or sepsis (*n* = 13), malignancy (n = 8), musculoskeletal disorders or fractures (*n* = 4), complications of arteriovenous access (*n* = 2), and others (*n* = 4). Table 4 shows the predictors for hospitalization. In the multivariate forward analysis, cerebrovascular disease, low albumin, high fasting glucose, high hemoglobin, high CaXP product and low LF (hazard ratios, 0.875; 95% CI, 0.814−0.941; *p* < 0.001) were independently associated with increased hospitalization.

**Table 4:** Predictors for hospitalization using Cox proportional hazards model

Outcome	Multivariate (forward)	
	Hazard Ratios (95% CI)	*p*
Hospitalization		
Cerebrovascular disease	5.673 (1.668–19.295)	0.005
Albumin (per 1 g/dL)	0.188 (0.064–0.551)	0.002
Fasting glucose (pre 1 mg/dL)	1.011 (1.002–1.019)	0.020
Hemoglobin (per 1 g/dL)	1.487 (1.127–1.962)	0.005
CaXP product (per 1 mg^2^/dL^2^)	1.034 (1.007–1.262)	0.015
LF (per 1 ms^2^)	0.875 (0.814–0.941)	< 0.001

Values expressed as Hazard Ratios and 95% confidence interval (CI). Abbreviations are the same as in Table 1.

Covariates in the multivariate model include age, sex, duration of dialysis, a history of smoking, hypertension, coronary artery disease and cerebrovascular disease, systolic and diastolic blood pressure, albumin, fasting glucose, triglyceride, total cholesterol, hemoglobin, creatinine, potassium, total calcium, phosphorous, CaXP product, iPTH, uric acid, Kt/V and HRV parameters before hemodialysis. Forward stepwise selection included variables with entry and removal probability < 0.05.

## DISCUSSION

The present study evaluated the relationship between UA and changes in HRV before and after HD in non-diabetic patients. The results showed that the patients with a higher UA level were associated with decreased HF% and LF/HF increase after HD. In addition, higher levels of potassium and total calcium were positively correlated with changes in HRV parameters before and after HD in the study patients.

The most important finding of the present study is that hyperuricemia contributed to a smaller increase in HRV after HD in non-diabetic patients. HRV is a non-invasive method that can be used to assess the autonomic nervous system. It represents beat-to-beat variability in heart rate, and it has successfully been used in patients undergoing chronic dialysis [19]. Abnormalities in HRV primarily reflect dysregulation between the sympathetic and parasympathetic nervous systems. An increasing number of studies have applied HRV frequency-domain analysis to assess the autonomic nervous system due to its non-invasiveness and easy applicability. Previous studies have reported that hyperuricemia is associated with low values of HRV parameters in pregnancy women and ageing population [16, 17]. Hyperuricemia has been associated with factors related to insulin resistance including obesity, dyslipidemia and hypertension [14, 20, 21]. Several mechanisms have been proposed for alterations in cardiac autonomic function underlying the pathogenesis of insulin resistance [22]. Sympathetic activation has been shown to inhibit insulin secretion from beta cells in the pancreas, leading to reduced glucose transport to peripheral tissues [23]. In addition, sympathetic vasoconstriction can cause a reduction in blood flow thereby reducing the uptake of glucose in skeletal muscles, and this can result in a reduction in the number of available capillaries and an increase in the distance that insulin has to travel to the cell membranes [22]. Moreover, sympathetic activation has been shown to increase adipose tissue lipolysis and the level of circulating free fatty acid [24]. This may then lead to an increase in insulin resistance and potentially hyperinsulinemia, and this can induce central sympathetic outflow resulting in a vicious circle [24]. The proposed association between hyperuricemia and autonomic dysfunction could be explained by the commonly seen insulin resistance in HD patients, which has been shown to be correlated with low HRV [25]. Moreover, we found an association between hyperuricemia and decreased ∆HRV after HD in our non-diabetic patients. Therefore, lowering UA levels may have the potential to improve increased HRV in non-diabetic HD patients. However, further studies are needed to confirm this hypothesis.

The second important finding of this study is that an increased potassium level was associated with increased changes in HRV parameters before and after HD. Membrane potential is primarily governed by the permeability of a membrane to potassium ions and the concentration gradient, and to some extent by the Na+/K+ pump. The potassium gradient plays a vital role in many physiological processes, including transmitting action potentials in nerve cells, maintaining cellular membrane potential, and cell volume homeostasis [26]. Higher levels of extracellular potassium can result in cell membrane potential depolarization through an increased potassium equilibrium potential. When the level of potassium exceeds a certain level, this depolarization opens potassium channels and inactivates sodium channels, thereby causing the cells to become refractory. This can then lead to neuromuscular, cardiac, and gastrointestinal organ system impairments, which in turn can cause ventricular fibrillation, an abnormally slow heart rhythm, and asystole [26]. Another possible explanation is that decreased serum potassium during HD is negatively correlated with the basal intracellular potassium concentration [26]. This loss of serum potassium then results in a lower threshold for cell depolarization, thereby increasing QT variation and inducing cardiac arrhythmia [27] which could theoretically affect HRV.

Zhang et al. [28] investigated the relationship between mineral metabolism and HRV in patients with stage 5 chronic kidney disease, and found significant associations between low HRV parameters and abnormal levels of serum calcium, iPTH and phosphorous, and that parathyroidectomy may reverse the risk of cardiovascular disease [28]. These findings suggest that both dysregulation of cardiovascular autonomic control and abnormal mineral metabolism may contribute to the higher risk of cardiovascular disease. In the present study, we found that a higher total calcium level was associated with greater changes in HRV parameters before and after HD. Therefore, a higher level of calcium may be associated with increased sympathetic activity, decreased arterial compliance and intradialytic hypertension [29, 30]. A higher calcium level has been reported to be associated with an increase in myocardial contractility [31], which may explain our findings.

There are several limitations to this study. First, the number of patients was relatively small, which may have affected the results. In future studies, we will enroll a larger number of patients. Second, this study was cross-sectional, and causal relationships could not be confirmed. In addition, heart rate autonomic modulation follows a circadian rhythm, with lower HRV during the day due to higher sympathetic activity, and higher HRV during the night due to vagal modulation. We performed all HRV examinations during the day (between 8 a.m. and 5 p.m.) to minimize the influence of the circadian rhythm, however we cannot rule out the possibility that this rhythm may not have been followed in all of the patients. Lastly, we did not evaluate the effect of anti-hypertensive medications on autonomic nerve function because this study was not a clinical trial aimed at investigating the effects of medications. However, the use of β-blockers, the most commonly used drug that can interfere with autonomic response, was not associated with any of our results.

In conclusion, our results showed that hyperuricemia contributed to a smaller increase in HRV after HD in patients without diabetes, reflecting a state of impaired sympatho-vagal equilibrium in non-diabetic patients with hyperuricemia undergoing HD. Lowering UA levels may therefore have the potential to improve increases in HRV in non-diabetic HD patients. In addition, high levels of potassium and total calcium were associated with greater changes in HRV after HD.

## MATERIALS AND METHODS

### Study patients and design

This study was conducted at a regional hospital in southern Taiwan. We included all patients undergoing maintenance HD except for those receiving HD during night shifts. In total, we enrolled 96 non-diabetic patients (35 males and 61 females) from May 2012 to July 2012. The study protocol was approved by our Institutional Review Board, and all of the enrolled patients provided written informed consent. All of the patients received HD three times per week, with each session lasting 3.5−4.5 hours with a blood flow rate of 250−300 mL/min and dialysate flow of 500 mL/min. Blood samples were taken before and after HD to calculate Kt/V.

### Electrocardiogram signal processing

All of the recruited subjects received short-term power spectral analysis of HRV, with all measurements being conducted in a quiet, temperature-controlled (28°C) room. HRV was analyzed according to standard methods. [32–34] A pericardial electrocardiogram (ECG) was performed continuously for 5 minutes with the patient lying quietly and breathing normally in the supine position for at least 10 minutes. The patients received these ECG examinations 30 minutes before and after the HD sessions, which were performed during the day (between 8 a.m. and 5 p.m.). ECG signals were recorded using an HRV analyzer (SS1C, Enjoy Research, Taipei, Taiwan) with an analog-to-digital converter and sampling rate of 256 Hz. Digitized ECG signals were analyzed online and stored on a computer for off-line verification. A computer program was used to identify each QRS complex, and ventricular premature complexes and noise were rejected according to a standard QRS template. Constant R-R values were measured again and interpolated at a rate of 7.11 Hz to achieve consistency in the time domain [33].

### HRV frequency-domain analysis

Non-parametric fast Fourier transformation (FFT) was used to analyze the frequency-domain. Following deletion of the direct current component, a Hamming window was used to reduce the effect of leakage [35]. Power spectrum density was estimated for each time segment (288 s; 2048 data points) using a computer algorithm based on FFT. Attenuation of this power spectrum caused by the sampling and Hamming window was then corrected, followed by quantification into standard frequency-domain parameters, [1] including LF (0.04–0.15 Hz), HF (0.15–0.40 Hz) and LF/HF HRV. LF and HF were normalized to the percentage of total power to detect any sympathetic influence on HRV as follows: LF% = LF/(total power-VLF [very low frequency])*100, and HF% = HF/(total power-VLF)*100. All of the HRV parameters were then logarithmically transformed to reduce skewness of distribution [1]. HF can be used to represent respiratory sinus arrhythmia and vagal control of heart rate [2]. Both vagal and sympathetic activity have been reported to contribute to LF HRV [3], and normalized values of LF (LF%) and the LF/HF ratio have been reported to reflect sympatho-vagal balance or sympathetic modulation [1]. Changes in HRV (∆HRV) were assessed before and after HD, and calculated as post-HD HRV minus pre-HD HRV.

### Collection of demographic, medical, and laboratory data

Demographic and medical data including age, gender and co-morbidities were obtained from the patients’ medical records and interviews. were measured from Fasting blood samples were taken for assessments of laboratory data, which were measured using an autoanalyzer (Roche Diagnostics GmbH, D-68298 Mannheim COBAS Integra 400). Concentrations of serum iPTH were measured using a two-sided immunoradiometric assay (CIS Bio International, Saclay, France). Kt/V was measured using the procedure described by Daugirdas as a marker of the efficiency of dialysis [36]. Data regarding the use of β-blockers, calcium channel blockers, ARBs, ACEIs and statins during the study period were also recorded from the patients’ medical records.

### Outcome of hospitalization

Clinical outcome of hospitalization was assessed. Model for hospitalization was censored when patients had hospitalization from any cause or at the end of the follow-up until June 2015.

### Statistical analysis

All statistical analyses were performed using SPSS version 17.0 for Windows (SPSS Inc., Chicago, USA). Data are expressed as percentage, mean ± standard deviation, or mean ± standard error of the mean for ∆HRV parameters, or median (25th–75th percentile) for the duration of dialysis and levels of triglycerides and iPTH. Differences between groups were assessed using the chi-square test for categorical variables, the independent *t*-test for continuous variables with an approximately normal distribution, or the Mann-Whitney *U* test for continuous variables with skewed distribution. Relationships between UA and ∆HRV parameters were assessed using bivariate correlations (Pearson’s correlation). ∆HRV parameters were defined as the HRV parameters measured after HD minus those measured before HD. Multiple stepwise linear regression analysis was used to identify the factors associated with the ∆HRV parameters. Multiple forward stepwise Cox proportional hazard analysis was used to identify the factors associated with hospitalization. A *p* value of less than 0.05 was considered to be statistically significant.

## Abbreviations

(HRV): heart rate variability
(HD): hemodialysis
(UA): uric acid
(HF): high frequency
(LF): low frequency
(iPTH): intact parathyroid hormone
(ACEIs): angiotensin converting enzyme inhibitors
(ARBs): angiotensin II receptor blockers
(ECG): electrocardiogram
(FFT): Fourier transformation
(VLF): very low frequency
